# Analysis of Antibiotic Consumption by AWaRe Classification in Shandong Province, China, 2012–2019: A Panel Data Analysis

**DOI:** 10.3389/fphar.2021.790817

**Published:** 2021-11-22

**Authors:** Jia Yin, Hongyu Li, Qiang Sun

**Affiliations:** ^1^ Center for Health Management and Policy Research, School of Public Health, Cheeloo College of Medicine, Shandong University, Jinan, China; ^2^ NHC Key Lab of Health Economics and Policy Research, Shandong University, Jinan, China

**Keywords:** antibiotics, consumption, health care institutions, antibiotic stewardship, China

## Abstract

**Introduction:** This study aims to examine the changes in trends and patterns of clinical consumption of antibiotics in Shandong, China based on Access, Watch, and Reserve (AWaRe) Classification after 10-years national antibiotic stewardship.

**Methods:** Antibiotic consumption data of all health care institutions for the period of 2012–2019 were obtained from the Drug Centralized Bidding Procurement System of Shandong. Shandong is a province that has the second-largest population in China. Five of the 16 cities in Shandong were high-income areas (HIAs) and the other 11 cities were grouped into upper-middle-income areas (UMIAs). The main outcome measures were the antibiotic consumption rates (DDD per 1,000 inhabitants per day, DID) and the proportions of different groups of antibiotics.

**Results:** The overall antibiotic consumption rate increased from 12.859–15.802 DID between 2012 and 2014, then continuously decreased to 9.771 DID in 2019. The consumption rate of access, watch, and reserve antibiotics have reduced since 2014 with a compound annual growth rate of −10.1, −9.0, and −8.1%, respectively. During 2012–2019, the access group proportion reduced from 50.0 to 44.9%, while the proportion of the watch group increased from 42 to 45.2%. The antibiotic consumption rate increased from 2012 to 2019 (from 7.38 to 9.12 DID) in the HIAs but sharply decreased in the UMIAs from 2014 to 2019 (from 17.94 to 10.05 DID). The watch antibiotics had the highest proportion of consumption in the HIAs (55.3% in 2019), while the access group had the highest proportion of consumption in the UMIA (49.5% in 2019).

**Conclusion:** The antibiotic stewardship policies launched in the last 10 years have contributed to reducing the clinical antibiotic consumption in Shandong. These policies have different effects on areas with different economic levels. The pattern of antibiotic consumption is still inappropriate in China as the watch group of antibiotics was consumed the most.

## Introduction

Since the first antibiotic was discovered over 100 years ago, antibiotics have saved thousands of lives suffered from infectious diseases (Hutchings et al., 2019). However, inappropriate- or over-prescription of antibiotics were widely reported all over the world and has been proved to be associated with the increasing prevalence of antimicrobial-resistance (AMR) strains (Goossens et al., 2005; Mendelson and Matsoso, 2015). AMR has gradually become a global public health crisis that affects human health, increases medical expenditures, and even hinders social and economic development (Bloom et al., 2017; World Bank, 2017). In 2017, to support antibiotic surveillance and stewardship at local, national and global levels, WHO developed the Access, Watch, and Reserve (AWaRe) Classification of antibiotics and updated it in 2019 (World Health Organization, 2019). The access group contains 48 antibiotics that are recommended as the first- or second-choice for treating common infectious syndromes, while the watch group includes 110 antibiotics that have higher resistance potential and are suggested to be used for specific infectious syndromes. There are 22 antibiotics in the reserve group, which are considered as the last resort of treatment. In the not recommended group, 103 fixed-dose combinations of multiple broad-spectrum antibiotics are listed and not recommended for use in clinical practice.

China has a huge pharmaceutical market. In 2020, the total terminal sales of pharmaceutical products reached 1708 billion RMB in China, with 69.4 and 30.6% sold in health care institutions [including public and private hospitals, and primary healthcare centres (PHCs)] and retail private pharmacies, respectively (Ministry of Commerce, 2021). China is also one of the countries that consumed the most antibiotics in the world, and has attracted more and more global attention on the overuse of antibiotics and AMR during the last decade (Heddini et al., 2009; Yezli and Li, 2012; Van Boeckel et al., 2014). Studies published before 2014 showed an overall antibiotic prescribing rate of 83.7% among outpatients with upper respiratory tract infections (URTI) in China (Li et al., 2016). To improve antibiotic stewardship and combat AMR, China began to explore strategies in 2011 in the context of the new health system reform (Yip et al., 2019). In 2012, the ministry of health released a mandatory regulation which was recognized as the strictest regulation for antibiotic use in China (Xiao and Li, 2013). It required the provinces to develop their own Antibiotic Classifications List (ACL) due to various antibiotic-use behaviors and bacterial resistance. Antibiotics are classified into non-restricted, restricted or special-grade in the ACL according to their effectiveness, safety, bacterial resistance and price. Physicians with junior professional titles who often work at the community level could only prescribe non-restricted antibiotics. For physicians with intermediate or senior professional titles who usually work in secondary or tertiary hospitals, they could prescribe all classes of antibiotics and non-restricted or restricted antibiotics, respectively. During 2011 and 2013, a special national campaign was implemented with the aim of promoting clinical use of antibiotics mainly in secondary and tertiary hospitals (Xiao, 2018). In 2014, a guiding policy was issued that emphasized the rational use of antibiotics in PHCs (National Health and Family Planning Commission, 2014). After 2015, more actions were taken at the national level. In 2016, 14 ministries joint-issued the “National Action Plan on antimicrobial resistance (2016–2020)” in response to WHO’s call (NHFPC and other 13 ministries, 2016). This action plan set out targets for AMR control during 2016 and 2020 and provided guidance on specific measures. From 2017 to 2020, guidance documents on improving clinical use of antibiotics and AMR surveillance in children were released (Children’s National Medical Center, 2018; National Health Commission, 2020a).

The current published studies conducted in China often focus on the patterns of clinical use of antibiotics in selected tertiary hospitals with findings that indicate a decline in antibiotic prescribing rates (Bao et al., 2015; Wushouer et al., 2017; Chen et al., 2018). Few studies analyzed the changes of antibiotic consumption using data collected from all the health care institutions in a provincial area (Lin et al., 2016; Yin et al., 2018; Xu et al., 2020). In context of China’s 10-years national antibiotic stewardship program, this study analyzed the changes in trends and patterns of antibiotic consumption in health care institutions in the second-largest population province of China, by WHO AWaRe Classification.

## Methods

### Study Design and Setting

This study used secondary data on antibiotic consumption collected from the Drug Centralized Bidding Procurement System of Shandong Province (referred as CBP system) over the period 2012–2019. Shandong is an eastern province of China traversed by the yellow river, with a population of more than 100 million in 2020. The gross national income (GNI) per capita in China and Shandong Province were $10224 and $10506 in 2019 ($1 equal to 6.8985 RMB in 2019), respectively. In 2020, the gross sales of pharmaceuticals in Shandong province ranks 6^th^ among the 34 provincial administrative districts in China (Ministry of Commerce, 2021). There are 16 cities in Shandong. According to the country classification defined by World Bank in 2021 (The World Bank, 2021), five cities were identified as high-income economies and were grouped into high-income areas (HIAs). The other 11 cities were identified as upper-middle-income economies and were grouped into upper-middle-income areas (UMIAs).

### Data Source and Analysis

The CBP system is an official online medicine trading platform that was established throughout the country in 2009. Health care institutions in Shandong were required to procure all medicines through the CBP system since the end of 2011. Consumption data of the private pharmacies is not available from this system. It records name of region, names of buyer and manufacturer, procurement date, receiving date, generic name of drug, strength, dosage and package, request quantity and unit price. Data of each record of antibiotic consumption between 2012 and 2019 was exported from this system into excel format, then cleaned and analyzed using Stata 16.0.

In China, health services are mainly provided by public health care institutions run by the government. In 2019, the total revenue of private health care institutions accounted for only 13% of all health care institutions (National Health Commission, 2020b). We divided the health care institutions into hospitals, PHCs, and others. Hospitals include secondary and tertiary hospitals. PHCs represent township hospitals and village clinics in rural areas and community health centres or stations in urban areas. Maternity and child healthcare hospitals, tuberculosis dispensary and private hospitals were classified into the group of others. According to the 2019 WHO AWaRe classification (World Health Organization, 2019), all the antibiotics consumed were classified into the categories of Access, Watch, Reserve, Not Recommended, and Unclassified.

All the procured antibiotics, including antibacterial for systemic use (J01), antimycotics for systemic use (J02), and intestinal anti-infectives (A07A), were standardized according to the Anatomical Therapeutic Chemical and Defined Daily Dose (ATC/DDD) system recommended by WHO (WHO Collaborating Centre for Drug Statistics Methodology, 2021). Antibiotic consumption was normalized using the number of DDD per 1,000 inhabitants per day (DID). The numbers of inhabitants were referred from the Statistical Year Reports of Shandong (2013–20). Compound Annual Growth Rate (CAGR) was used to calculate the yearly changes in antibiotic consumption.

## Results

**FIGURE 1:**
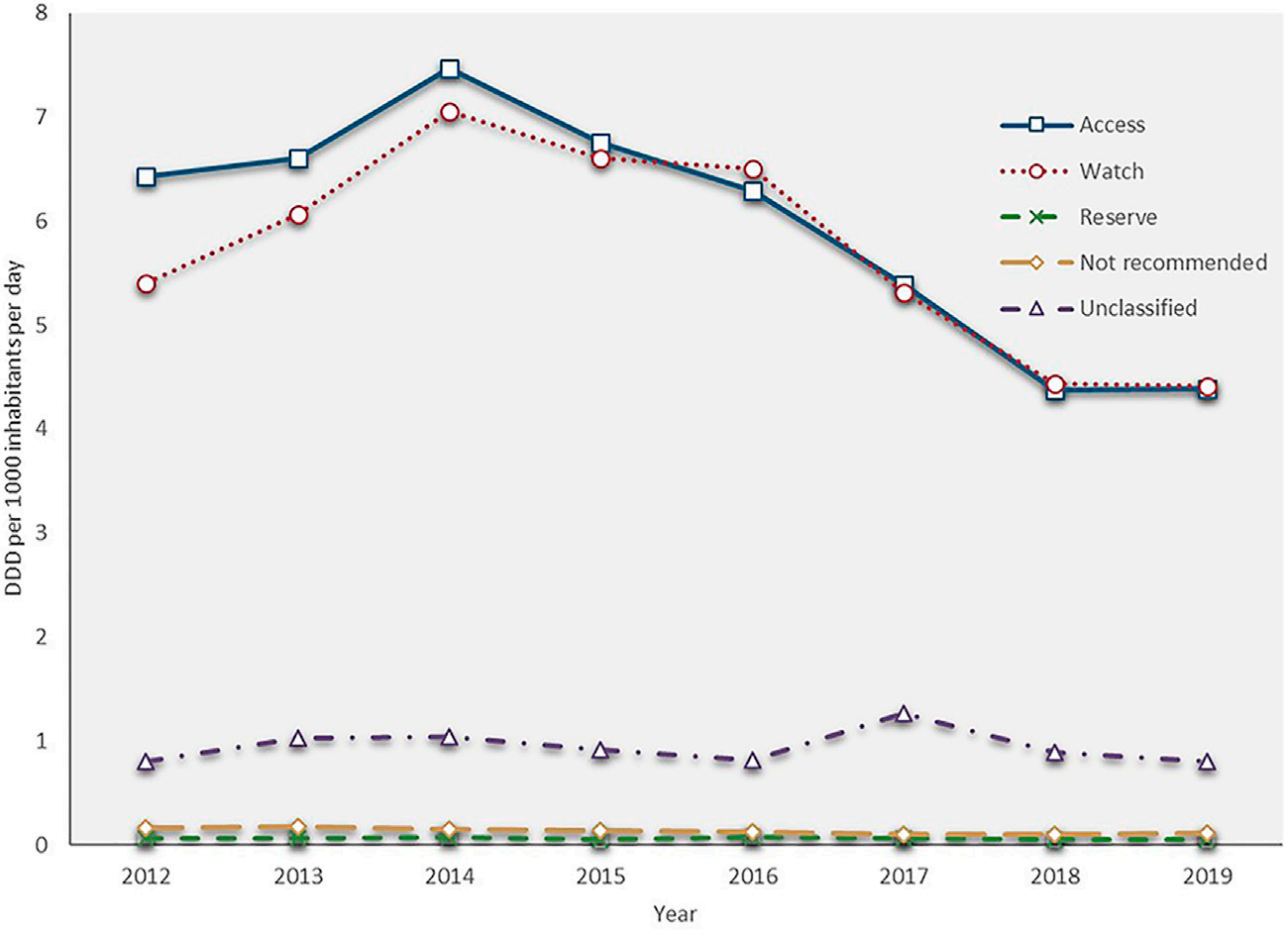
Antibiotic consumption by AWaRe Classification, 2012–19.

**FIGURE 2:**
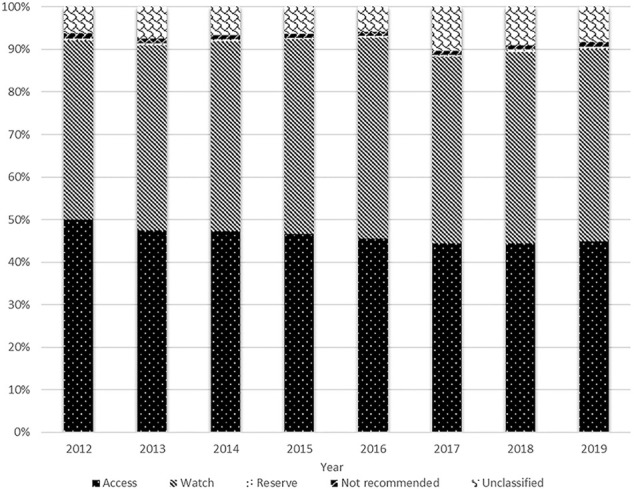
Distribution of antibiotic consumption by AWaRe Classification, 2012–19.

The total antibiotic consumption rate increased by 22.9%, from 12.859 DID in 2012 to 15.802 DID in 2014, then continuously reduced at a CAGR of −9.2% between 2014 and 2019 (9.771 DID in 2019). The consumptions of the access, watch and reserve antibiotics also reached their peaks in 2014 (7.468, 7.059 and 0.077 DID), then all kept decreasing during 2014 and 2019 with a GACR of -10.1%, −9.0% and −8.1%, respectively (4.384, 4.409. and 0.050 DID in 2019) (Figure 1). The consumption of the access group accounted for less than 50% of the total and this proportion continuously decreased from 50.0 to 44.9% over the study period. However, the proportion of watch antibiotic consumption kept increasing and exceeded the proportion of the access group in 2016, then slightly reduced from 47.1% in 2016 to 45.2% in 2019. The consumption of reserve antibiotics accounted for around 0.5% of the total during the study period (Figure 2).

**FIGURE 3:**
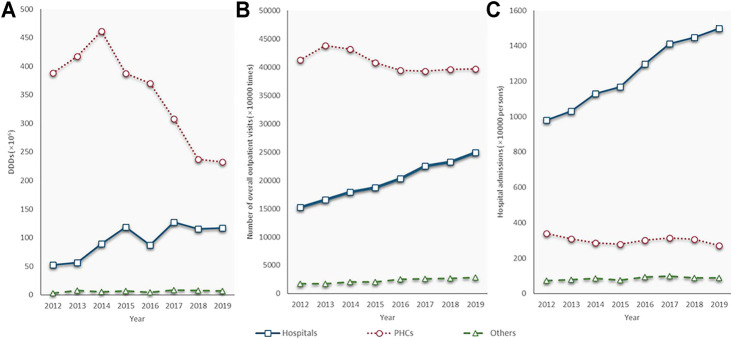
Antibiotic consumption and quantity of health service utilization by types of health care institutions, 2012–19. **(A)** Antibiotic consumption; **(B)** Number of outpatient visits; **(C)** Number of admissions; PHC: primary health care centre; Source of **(A)** and **(B)**: Shandong bulletin of health development, 2013–2020.

The PHC was the leading antibiotic consumer. In 2012, the PHCs consumed 7.4 times as much volume of antibiotics as hospitals (388.4 million DDDs vs. 52.3 million DDDs). From 2014 to 2019, the PHCs saw a sharp fall, from 461.5 to 232.3 million DDDs, at a CAGR of −12.8%. Alternatively, the volume of antibiotics consumed by hospitals, although slightly declined in 2016, has doubled in 2019 (117.2 million DDDs) compared with that in 2012 (52.3 million DDDs). Health service utilization showed similar trends as antibiotic consumption in both the hospitals and PHCs. Outpatient visits and admissions increased at a GACR of 7.3 and 6.3% in hospitals but decreased with a GACR of 0.58 and 3.15% in the PHCs between 2012 and 2019. The consumption of the other group fluctuated during the study period, and it only accounted for 0.8% of the total on average (Figure 3).

**FIGURE 4:**
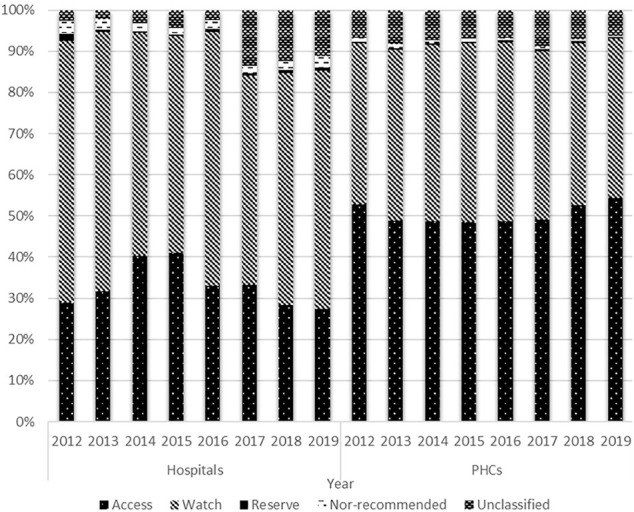
Distribution of antibiotic consumption by AWaRe Classification and types of health care institutions, 2012–19.

In hospitals, access antibiotic consumption accounted for about one-third of the total antibiotic consumption. The proportion of access antibiotic consumption decreased yearly since 2015, while the proportion of watch antibiotic consumption maintained at 50–60%. In 2019, the volume of access antibiotics was only about one-half of the volume of the watch antibiotics in hospitals. In the PHCs, the proportions of the access and watch antibiotic consumptions were about 50 and 40%, respectively. The proportion of access antibiotic consumption has been increasing since 2015. By contrast, the proportion of the watch antibiotics consumed in the PHCs tended to decline between 2015 and 2019 (Figure 4).

**FIGURE 5:**
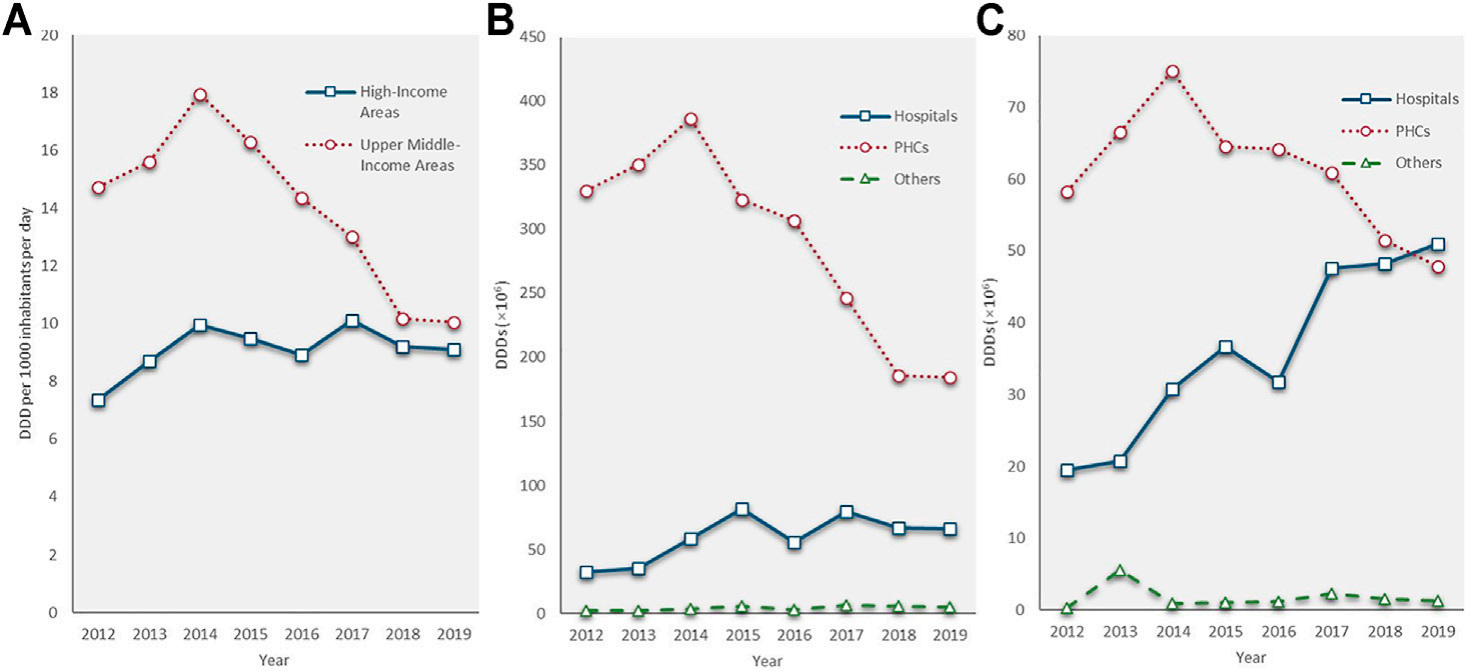
Antibiotic consumption by economic level of areas, 2012–19. **(A)** Total antibiotic consumption; **(B)** Antibiotic consumption in upper-middle-income areas; **(C)** Antibiotic consumption in high-income areas.

People living in the UMIAs consumed twice as much antibiotics as people living in the HIAs between 2012 and 2014 (UMIAs: 14.74–17.94 DID; HIAs: 7.38–9.96 DID). The antibiotic consumption rate in the UMIAs having sharply declined at a CAGR of −10.9% since 2014 (17.94 DID) and reached a rate (10.05 DID) close to that in the HIAs (9.12 DID) in 2019. In the HIAs, the antibiotic consumption rate fluctuated slowly from 9.96 DID in 2014 to 9.12 DID in 2019. In both economic-level areas, the volume of antibiotic consumption showed a decreasing trend in the PHCs, while an increasing trend in hospitals. In the HIAs, the volume of antibiotics consumed by hospitals grew at a faster rate (CAGR: 14.7%) than that in the UMIA (CAGR: 10.6%) during the last 8 years and surpassed the volume of antibiotics consumed by the PHCs in 2019 (Figure 5).

**FIGURE 6:**
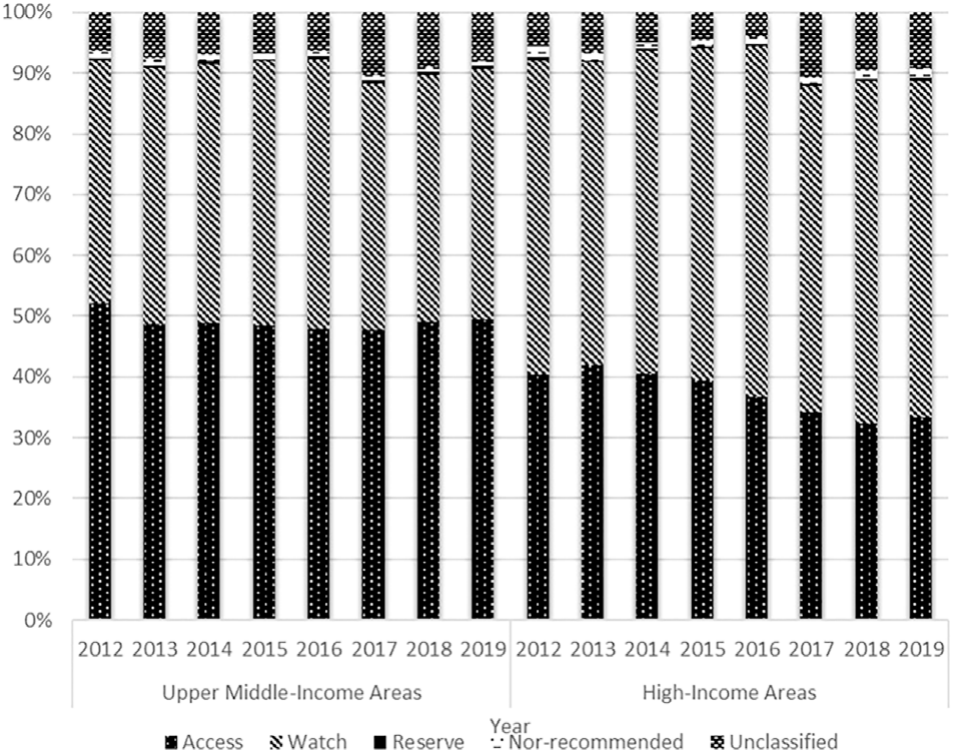
Distribution of antibiotic consumption by AWaRe Classification and economic level, 2012–19.

In terms of access antibiotic consumption, the proportion maintained at approximately 50% in the UMIAs (2012: 52.0%; 2019: 49.5%), while decreasing yearly from 41.9 to 33.3% in the HIAs during the study period. The proportion of watch antibiotic consumption showed a trend of growth between 2012 and 2016 (HIAs: 51.7–57.7%; UMIAs: 39.9–44.4%) and then declined between 2016 and 2019 (HIAs: 57.7–55.3%; UMIAs: 44.4–41.2%) in both economic-level groups. With quite a small proportion (<1%) in both groups, the reserve antibiotics started to grow in 2015 and doubled in 2019 compared with 2015 (0.69 vs. 0.33%) in the HIAs (Figure 6).

In terms of the total consumption, the top three consumed access antibiotics were all penicillins, including amoxicillin (J01CA04), amoxicillin and beta-lactamase inhibitor (J01CR02), and benzylpenicillin (J01CE01). Amoxicillin was also the most consumed antibiotic among all, the consumption of which accounted for 27.2% of the total volume in 2019. Only amoxicillin and beta-lactamase inhibitor showed an increasing trend, while all the other access antibiotics declined over the past four or 5 years. The most commonly consumed watch antibiotic was levofloxacin (J01MA12), followed by cefuroxime (J01DC02) and roxithromycin (J01FA06). All the watch antibiotics showed a downward trend. In the reserve antibiotic group, fosfomycin (J01XX01), minocycline (J01AA08), and linezolid (J01XX08) were the most commonly consumed. Three of the top five consumed reserve antibiotics, which were minocycline (J01AA08), linezolid (J01XX08), and tigecycline (J01AA12) had growth trends in recent years (Table 1).

**TABLE 1:** Top five antibiotics in consumption rate by AWaRe Classification, 2012–19.

AWaRe classification	ATC code	Antibiotics	DID							
			2012	2013	2014	2015	2016	2017	2018	2019
Access	J01CA04	Amoxicillin	2.568	1.918	3.122	3.018	3.076	2.750	2.488	2.657
	J01CR02	Amoxicillin and beta-lactamase inhibitor	0.318	0.364	0.465	0.463	0.518	0.501	0.476	0.531
	J01CE01	Benzylpenicillin	1.047	1.214	0.965	0.731	0.630	0.469	0.371	0.313
	J01DB01	Cefalexin	0.936	1.228	0.982	0.787	0.756	0.484	0.278	0.263
	J01GB03	Gentamicin	0.014	0.015	0.417	0.401	0.362	0.191	0.118	0.108
Watch	J01MA12	Levofloxacin	0.673	0.872	1.194	1.152	1.131	0.876	0.764	0.808
	J01DC02	Cefuroxime	0.332	0.463	0.938	0.896	0.752	0.667	0.581	0.585
	J01FA06	Roxithromycin	0.933	1.191	1.091	0.909	0.967	0.709	0.578	0.515
	J01FA10	Azithromycin	0.479	0.530	0.542	0.534	0.603	0.485	0.383	0.396
	J01DD04	Ceftriaxone	1.041	1.199	1.177	1.061	1.068	0.642	0.371	0.351
Reserve	J01XX01	Fosfomycin	0.038	0.052	0.068	0.024	0.046	0.043	0.035	0.029
	J01AA08	Minocycline	0.001	0.001	0.002	0.003	0.005	0.005	0.008	0.012
	J01XX08	Linezolid	0.001	0.001	0	0.001	0.001	0.002	0.003	0.004
	J01DF01	Aztreonam	0.023	0.006	0.005	0.016	0.018	0.011	0.006	0.002
	J01AA12	Tigecycline	0	0	0	0	0	0.001	0.002	0.002

## Discussion

This study used antibiotic procurement data of almost all the health care institutions in a large-population province of China from 2012 to 2019. China has taken special actions to reduce antibiotic use and to combat AMR since 2011. After a decade of national antibiotic stewardship, the consumption of access, watch, and reserve antibiotics in health care institutions in Shandong Province have all showed decreasing trends since 2015. However, the access antibiotics had a greater reduction than the watch antibiotics. This resulted in a lower proportion of access antibiotics. The reduction in total consumption was mainly attributed to the remarkable decrease in antibiotic consumption at the PHCs and the UMIAs.

The antibiotic consumption rate in Shandong is at a lower level among the 34 provincial administrative districts in China and is also lower than the global median (17.2 DID in 2015) (Wushouer et al., 2020; Klein et al., 2021). The group of access antibiotics, as the first choice of treatment for common infections, was higher in Shandong than the national average in terms of the consumption rate and the proportion of the total, but lower than in most of the other countries (Wushouer et al., 2020). The national antibiotic stewardship strategies appear to play a role in reducing antibiotic use at health care institutions in Shandong Province, as well as in Hubei Province and Shanghai Municipality (Lin et al., 2016; Zhang et al., 2019). However, other studies indicate that China’s national antibiotic stewardship strategies have had no significant effect on antibiotic consumption in Shanxi Province where the volumes of antibiotic consumption kept increasing between 2016 and 2018 (Xu et al., 2020).

Although China’s antibiotic stewardship policies was mainly targeted at hospitals, the volume of antibiotic consumption still showed an increasing trend in secondary and tertiary hospitals. On the contrary, the consumption of antibiotics at PHCs in Shandong dramatically declined in the last few years. In addition to the policy effect, this may be because China’s PHCs are shrinking while hospitals are expanding. According to the China Health Statistics Yearbook 2020 (National Health Commission, 2020b), the number of health care workers in township hospitals has reduced by 23%, and the health care utilization at PHCs continuously decreased from 2012 to 2019. However, the numbers of health care workers and beds in hospitals have increased by 60 and 65% during the same period. For one reason, the number of rural PHCs accounted for 80% of the total PHCs (National Health Commission, 2020b). Rural-to-urban migration in China has contributed to the reduction in the scale of rural PHCs. Another reason is that China has implemented the Hierarchical Medical System in 2015 to divert patients with common diseases to the PHCs, but patients could freely choose which health care institutions to visit, and they still prefer to visit hospitals due to the higher quality of health care (Zhou et al., 2021). Moreover, PHCs mainly provide outpatient services, which are typically not covered by the basic medical insurance schemes. Thus, residents are more likely to visit hospitals for inpatient services that could be reimbursed.

Overall, there was no obvious change in the proportions of watch antibiotics. The proportion of watch antibiotics was much higher than that of the access antibiotics in the hospitals. However, this proportion decreased and was much lower than that of access antibiotics in the PHCs. This decrease may more likely be related to the effects of regulation which limited the prescription of antibiotics in watch and reserve groups by junior physicians. Therefore, some patients may visit hospitals instead of PHCs for restricted antibiotics. It should be noticed that the classifications of antibiotics in the ACL of Shandong, as well as the other provinces in China is different from those in the AWaRe Classification. All the top five watch antibiotics are grouped into non-restricted antibiotics in the Shandong ACL (2021 version), which may result in a lower policy impact on the watch group than the access one.

Globally, the high-income economies consumed more antibiotics per person than the low-or middle-income economies and consumed much more volume of access antibiotics than the watch antibiotics during 2011 and 2015 (Jackson et al., 2019). However, our study showed opposite results. The antibiotic consumption rate was lower in the HIAs than in the UMIAs. In the HIAs of Shandong, the watch group accounted for a higher proportion than the access group. Given that the two economic-level areas have the same political and cultural circumstances, it may reflect the importance of knowledge and attitudes of patients and health providers towards the rational use of antibiotics. In China, inappropriate use of antibiotics was more serious in less developed areas, especially in the rural areas (Shen et al., 2021). Thus, there was a greater improvement in the antibiotic consumption rate in the UMIAs, driven by antibiotic stewardship policies. Moreover, the HIAs have well-known hospitals that attract patients with serious diseases. This may cause a higher proportion of watch antibiotic consumption in these areas.

Although the consumption rate of reserve antibiotics also decreased in Shandong, it has the lowest reduction rate among all three categories. Linezolid (J01XX08) and tigecycline (J01AA12), two relatively new antibiotics that are often used to treat severe infections caused by multi-drug resistant bacteria (Kemung et al., 2018), had increasing consumption rates in Shandong. This may indicate that antibiotic-resistant infections are on the rise in hospitals.

### Strengths and Limitations

This study has several strengths. First, our study analyzed antibiotic procured by almost all the health institutions in a large-population province in China. Currently, there is no national data analysis on antibiotics consumed by all health care institutions because the CBP system is established at the provincial level and the recorded data is not shared with the other provinces. Since there were few studies focused on antibiotic consumption over the whole province, this study will add a big piece of the puzzle regarding China’s antibiotic consumption. Second, the study period was during 2012 and 2019 when China began to intensively issue antibiotic stewardship policies. Our findings showed a decreasing trend of antibiotic consumption rate, which may likely reflect the effect of the national policies. These policies could be tailored to other countries or areas with similar circumstances. Third, by analyzing the pattern of antibiotic consumption according to AWaRe Classification by regional economic level and types of health care institutions, our study could explore the priorities for future antibiotic governance.

Nevertheless, this study is limited by the observational study design and could not prove the causal relationship between policies and changes in antibiotic consumption. Further, because China has launched a series of policies to improve antibiotic use and to control AMR in the last 10 years, it is likely impossible to identify the effect of a single policy. Another limitation is that our study lacked antibiotic consumption data from private pharmacies. According to a study conducted in private pharmacies of China using a simulated client method, 70% adult URTI visits were dispensed with non-prescription antibiotics in 2017 (Chang et al., 2019). However, the sales of antibiotics from private pharmacies accounted for less than one-third of the total sales of medicines in China (Ministry of Commerce, 2021). Additionally, China has committed to reduce the non-prescription sale of antibiotics in private pharmacies. Early in 2009, a policy which requires patients to procure Rx drugs with prescriptions at private pharmacies and private pharmacies to hire licensed pharmacists who are responsible for reviewing the legality and rationality of prescriptions was released by the Ministry of Health (Minsity of Health and other 9 ministries, 2009). One of the targets of the 2016–2020 action plan is to prohibit antibiotic dispensing without prescription at all private pharmacies (NHFPC and other 13 ministries, 2016). Between 2011 and 2017, a decreasing trend of non-prescription antibiotic dispensing rate in private pharmacies was observed (Chang et al., 2019). Thus, the results of this study are believed to represent the majority of the total consumption of human-use antibiotics in Shandong. While the national policies were implemented across the country simultaneously, there are regional disparities in terms of economic, population, and health input that may influence antibiotic consumption. Therefore, future research should analyze China’s antibiotic stewardship policies using integrated national data to explore the potential reasons for the variability of policy effects in different provinces of China.

## Conclusion

The antibiotic consumption rate in health care institutions has indicated a downward trend in China’s Shandong Province after nearly a decade of antibiotic stewardship. However, the pattern of antibiotic consumption is still inappropriate in terms of the watch group of antibiotics consumed. In addition, the trends for antibiotic consumption were found to vary across areas with different economic levels. Indicating that further investigation is needed to understand the reasons for this variability in policy effects.

## Data Availability

The original contributions presented in the study are included in the article/Supplementary Material, further inquiries can be directed to the corresponding author.
